# L-EGCG-Mn nanoparticles as a pH-sensitive MRI contrast agent

**DOI:** 10.1080/10717544.2020.1862363

**Published:** 2020-12-26

**Authors:** Jiali Li, Xue Jiang, Lihuan Shang, Zhen Li, Conglian Yang, Yan Luo, Daoyu Hu, Yaqi Shen, Zhiping Zhang

**Affiliations:** a Department of Radiology, Tongji Hospital, Tongji Medical College, Huazhong University of Science and Technology, Wuhan, China; b Guangdong Provincial Key Laboratory of Malignant Tumor Epigenetics and Gene Regulation, Medical Research Center, Sun Yat-sen Memorial Hospital, Sun Yat-sen University, Guangzhou, China; c Tongji School of Pharmacy, Huazhong University of Science and Technology, Wuhan, PR China; d National Engineering Research Center for Nanomedicine, Huazhong University of Science and Technology, Wuhan, PR China; e Hubei Engineering Research Center for Novel Drug Delivery System, Huazhong University of Science and Technology, Wuhan, PR China

**Keywords:** MRI contrast agent, nanoparticle, manganese, EGCG, pH sensitivity

## Abstract

This study aimed to synthesize and characterize L-epigallocatechin gallate (EGCG) complexed Mn^2+^ nanoparticle (L-EGCG-Mn), a proof-of-concept pH-sensitive manganese core nanoparticle (NP), and compare its magnetic resonance (MR) properties with those of Gd-DTPA, both *in vitro* and *in vivo*. Reverse microemulsion was used to obtain the L-EGCG-Mn NPs. The physicochemical properties of L-EGCG-Mn were characterized using dynamic light scattering, transmission electron microscopy, and near-infrared fluorescence small animal live imaging. The *in vitro* relaxivity of L-EGCG-Mn incubated with different pH buffer solutions (pH = 7.4, 6.8, 5.5) was evaluated. The T1-weighted MR imaging (MRI) properties were evaluated *in vitro* using hypoxic H22 cells as well as in H22 tumor-bearing mice. Cytotoxicity tests and histological analysis were performed to evaluate the safety of L-EGCG-Mn. L-EGCG-Mn showed good biocompatibility, stability, pH sensitivity, and tumor-targeting ability. Moreover, when the pH was decreased from 7.4 to 5.5, the *r*_1_ relaxivity of L-EGCG-Mn was shown to gradually increase from 1.79 to 6.43 mM^−1^·s^−1^. Furthermore, after incubation with L-EGCG-Mn for 4 h, the T1 relaxation time of hypoxic H22 cells was significantly lower than that of normoxic H22 cells (1788 ± 89 vs. 1982 ± 68 ms, *p*=.041). The *in vivo* analysis showed that after injection, L-EGCG-Mn exhibited a higher MRI signal compared to Gd-DTPA in H22 tumor-bearing mice (*p* < .05). Furthermore, L-EGCG-Mn was found to have a good safety profile via cytotoxicity tests and histological analysis. L-EGCG-Mn has a good safety profile and pH sensitivity and may thus serve as a potential MRI contrast agent.

## Introduction

Cancer cells rely on the ‘Warburg effect’ for aerobic glycolysis, leading to the accumulation of high lactate concentrations, even under aerobic conditions (Peng et al., 2019). Although the presence of lactate in the tumor microenvironment (TME) was previously considered as metabolic waste, more recently, accumulating evidence has suggested that it acts as an important signaling molecule in the regulation of tumor metabolism and immunity (Zhang et al., 2019). Moreover, the lactate in the TME controls multiple phenomena associated with tumor resistance to therapy (Pilon-Thomas et al., 2016; Ippolito et al., 2019). Thus, the noninvasive detection of tumor acidic regions is critical not only for personalized medicine but also for prognosis prediction. The physiological pH of normal tissues and body fluids (including blood) is approximately neutral (7.35–7.45), whereas the pH of tumor tissues is more acidic (6.5–7.0), which decreases even further in hypoxic regions *in vivo* (<6.5) (Neri & Supuran, 2011). Engineered nanoparticles (NPs) that respond to acidic pH (<6.5) and release paramagnet components are expected to reflect tumor lactate level via magnetic resonance imaging (MRI) (Garcia-Hevia et al., 2019).

The earliest and most frequently used MRI contrast agent (CA) approved for clinical use was Gd^3+^-complexes. However, the safety of Gd-DTPA-BMA (gadodiamide) has become increasingly controversial since 2006 (Grobner, 2006; Marckmann et al., 2006). Currently, in addition to two hepatocyte-specific CAs, European countries ban the use of Gd-BOPTA (gadobenate dimeglumine), Gd-DTPA-BMA, Gd-DTPA (gadopentetate dimeglumine), and Gd-DTPA-BMEA (gadoversetamide) (Dekkers et al., 2018). Mn, an essential trace element in the human body, was shown to have a short T1 effect due to its five unpaired electrons (Reddi et al., 2009; Pan et al., 2011). Hence, Mn^2+^-based CAs have become of high interest for the development of novel MRI CAs (Gale et al., 2015; Erstad et al., 2019). In the last decade, scientists have developed numerous Mn-based NPs for tumor-specific MRI. Nevertheless, in the case of some CAs (Shin et al., 2009; Huang et al., 2010a, 2010b), the Mn^2+^ was trapped/coordinated in the NP, resulting in lower relaxation efficiency compared to free Mn^2+^. Recently, intelligent NPs (Cai et al., 2015; Mi et al., 2016; Li et al., 2017; Wang et al., 2018), which respond to the acidic conditions in tumor tissues, have been designed to improve the accuracy and sensitivity of imaging techniques by increasing the contrast between the tumor tissue and background. For example, one strategy involves Mn^2+^ doping into calcium phosphate (CaP) (Mi et al., 2016) or silica (Kim et al., 2013) to form pH-sensitive CAs. However, the inevitable degradation problem of these inorganic agents appears to limit further clinical applications (Fu et al., 2019). In contrast, epigallocatechin gallate (EGCG), an organic green tea extract with excellent antioxidant activity, has gained increasing attention in the biomedical field due to its good biocompatibility, pH sensitivity, and versatile functionalization capabilities (Reygaert, 2014, 2018). For example, NPs coordinating EGCG with metal ions, such as Fe^3+^ (Xiao et al., 2015), Cu^2+^ (Tsai et al., 2016), Au^3+^ (Jiang et al., 2019), or Sm^3+^ (Li et al., 2019), have been used to diagnose and treat tumors and have had impressive safety assessments. However, there are few studies on the chelation of EGCG with Mn^2+^ for MRI.

**Figure 1.**
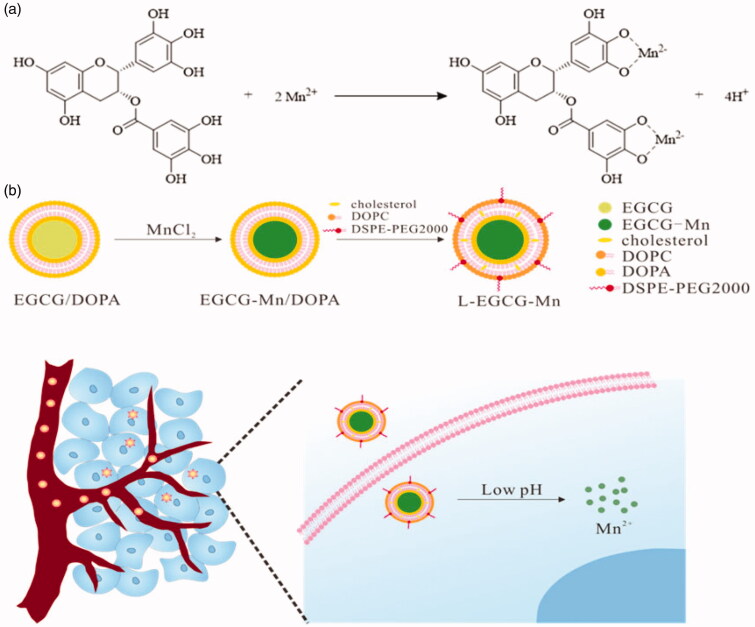
A scheme indicating the synthesis of L-EGCG-Mn NPs and the subsequent pH-sensitive mechanism *in vivo*. (a) Mn^2+^ coordinated with EGCG to form EGCG-Mn complexes. (b) The preparation of L-EGCG-Mn NPs and the mechanism of action of L-EGCG-Mn NPs *in vivo*.

As such, in this study, we fabricate a novel NP based on the chelation effect of EGCG and Mn^2+^ and obtained NPs that might be used as an MRI CA (Figure 1). The coordination interaction between the metal and EGCG was evaluated by previous studies (Rahim et al., 2018; Wang et al., 2018). Moreover, PEGylation was shown to enhance the stability of L-epigallocatechin gallate (EGCG) complexed Mn^2+^ nanoparticle (L-EGCG-Mn) and prolong circulation time (Li et al., 2010; Calcagno et al., 2019). In addition, the chelation of EGCG to Mn^2+^ would be weakened in an acidic environment (Navarro et al., 2005), thereby accelerating the release of Mn^2+^. Thus, L-EGCG-Mn could be disintegrated in a low pH environment to accurately control Mn^2+^ release and simultaneously present high relaxivity.

This study aimed to synthesize and characterize the pH-sensitive L-EGCG-Mn as well as verify its magnetic resonance (MR) properties, both *in vitro* and *in vivo*. The first commercially approved Gd chelate, Gd-DTPA, was used as the reference standard for MRI CA.

## Materials and methods

**Figure 2.**
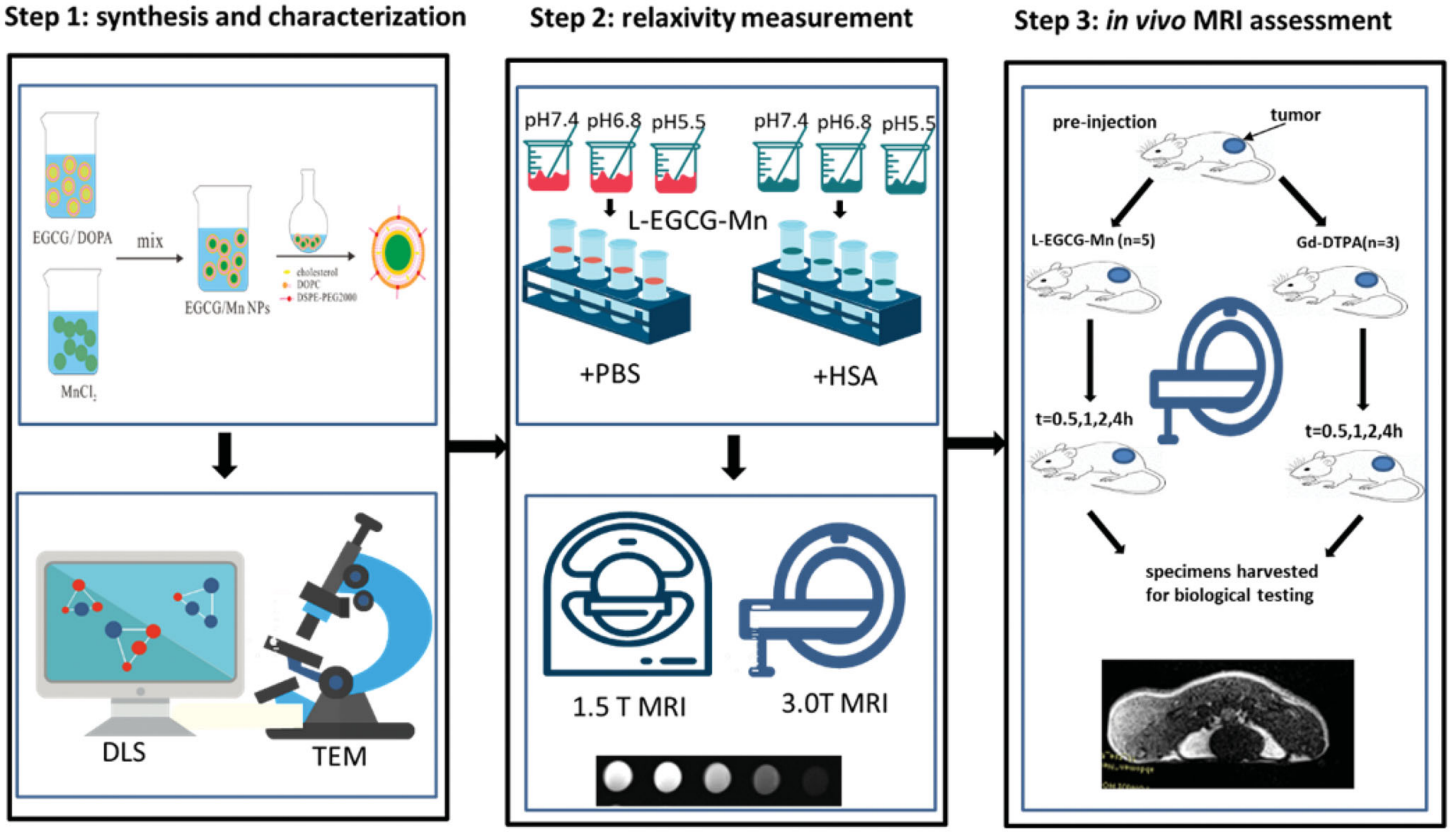
Flowchart of the entire experimental design. Step 1, synthesis and characterization. The reverse microemulsion method was used to obtain L-EGCG-Mn NPs. Step 2, relaxivity measurement. In each set of L-EGCG-Mn solution, the Mn concentration used was 0.04, 0.08, 0.2, 0.4, and 0.8 mM, respectively. Three samples per concentration were analyzed. Step 3, *in vivo* MRI assessment. Eight mice who received H22 cell transplantation were imaged using a 3 T MRI scanner (*n* = 5 injected with L-EGCG-Mn NPs, 6.4 μmol/kg Mn; *n* = 3 injected with Gd-DTPA, 6.4 μmol/kg Gd).

This study was approved by the local Ethics Committee and all experiments in this study strictly followed the Institutional Guidelines of Experimental Animal Care and Use. The entire workflow of this study is briefly shown in Figure 2.

### Materials

Manganese chloride (MnCl_2_), cyclohexane, and cobalt (iii) chloride hexahydrate (CoCl_2_) were purchased from Aladdin^®^ (Shanghai, China). EGCG was obtained from Purify^®^ (Chengdu, China) and IGEPAL CO-520 was obtained from Sigma-Aldrich (St. Louis, MO). Dioleoyl phosphatidic acid (DOPA), 1,2-dioleoyl-snglycero-3-phosphocholine (DOPC), cholesterol, and 1,2-dioleoyl-sn-glycero-3-phosphoethanolamine-N-[methoxy(polyethylene, glycol)-2000] (DSPE-PEG2000) were purchased from Avanti Polar Lipids, Inc. (Alabaster, AL), whereas 1,1′-dioctadecyl-3,3,3′,3′-tetramethylindotricarbocyanine iodide (DiR) was purchased from AAT Bioquest, Inc. (Sunnyvale, CA). The 3-(4, 5-dimethyl-thiazol-2-yl)-2,5-diphenyltetrazolium bromide (MTT) was obtained from BioSharp (Seoul, South Korea).

### Preparation of L-EGCG-Mn NPs

EGCG-Mn/DOPA NPs (EGCG-Mn NPs) were prepared using the reverse microemulsion method (Zhuang et al., 2016). Phase A, consisting of 100 μL of 10 mM EGCG and 100 μL of DOPA, was added to 4 mL CO-520/cyclohexane, and phase B, consisting of 100 μL of 80 mM MnCl_2_ was added to 4 mL CO-520/cyclohexane, and stirred separately for 0.5 h to form a reverse water-in-oil microemulsion. Next, phase A was added dropwise to phase B while stirring. After 2 h, 8 mL of ethyl alcohol was added to break the microemulsion. The mixture was then collected and centrifuged at 13,000×*g* for 15 min. Thereafter, the precipitate was washed twice with ethyl alcohol and dried using N_2_.

The L-EGCG-Mn NPs were prepared by dissolving EGCG-Mn/DOPA, 80 μL of 20 mM DOPC, 80 μL of 20 mM cholesterol, and 20 μL of 20 mM DSPE-PEG2000 in 4 mL trichloromethane. Next, trichloromethane was removed via rotary evaporation. DiR-labeled L-EGCG-Mn was prepared by dissolving EGCG-Mn/DOPA, DiR, DOPC, cholesterol, and DSPE-PEG2000 in trichloromethane, and the trichloromethane was removed with rotary evaporation. The L-EGCG-Mn NPs or DiR-labeled L-EGCG-Mn were then hydrated using phosphate-buffered saline (PBS) and incubated in a water bath at 37 °C for 0.5 h and then used for further applications.

### Characterization of the L-EGCG-Mn NPs

Zeta potential and particle size were measured via dynamic light scattering (DLS, ZetaPlus, Brookhaven Instruments, Holtsville, NY). Their morphology was evaluated through transmission electron microscopy (TEM, HITACHI H-7000 FA, Chiyoda City, Japan, acceleration voltage = 100 kV). The stability of the L-EGCG-Mn NPs was investigated by dispersing the L-EGCG-Mn NPs in PBS and fetal bovine serum (FBS). Thereafter, the change in particle size was recorded continuously for 1 week.

### Cell lines and tumor model

H22 and L929 cells were purchased from the Chinese Academy of Sciences Cells Bank (Shanghai, China). Cells were maintained in Dulbecco’s Modified Eagle Medium (L929) or Roswell Park Memorial Institute-1640 medium (H22) supplemented with 10% FBS and 1% penicillin and streptomycin under a humidified atmosphere (37 °C, 5% CO_2_). KM mice (female, 18–20 g; 4–5 weeks of age) were purchased from the local institutional animal care center and were acclimated to the center environment before study initiation.

After resuscitation, H22 cells were injected into the peritoneal cavity of KM mice. Carcinoma ascites were collected after seven days. The concentration of H22 cells was adjusted to 2 × 10^6^ cells/mL, and 100 μL of the H22 cell suspension was subcutaneously injected into the left side of the mice back to generate the tumor model (Bao et al., 2016).

### *In vitro* cytotoxicity evaluation

Cytotoxicity was evaluated using the MTT assay. Briefly, normal fibroblasts cells L929 were seeded in 96-well plates at a density of 8000 cells/well and cultured for 24 h. Next, the supernatant was removed and replaced with 100 μL of blank medium supplemented with various concentrations of L-EGCG-Mn NPs. After incubating for another 24 h, 10 μL of MTT (5 mg/mL) was added and the cells were then incubated for another 2 h. The liquid from each well was removed and replaced with 150 μL of dimethyl sulfoxide. The absorbance at 490 nm was detected using a microplate reader (Multiskan MK3, Thermo Fisher Scientific, Waltham, MA).

### Relaxivity measurement

#### Preparation of samples

Three sets of L-EGCG-Mn buffer solutions with different pH values (pH = 7.4, 6.8, and 5.5 PBS) were prepared. The Gd-DTPA in PBS (pH = 7.4) solution was used as a control. For each set of the L-EGCG-Mn solutions, the Mn concentrations used were 0.04, 0.08, 0.2, 0.4, and 0.8 mM. Likewise, the Gd concentration in the Gd-DTPA solution was 0.04, 0.08, 0.2, 0.4, and 0.8 mM. Three samples per concentration were analyzed. Thus, this group contained a total of 60 samples.

Forty-five more samples were prepared in the same manner and incubated with human serum albumin (HSA, 10 mg/mL) for 24 h. Fresh samples were prepared before the MR scan. Finally, 210 samples were analyzed via MR scanning (1.5 and 3 T at 22 °C).

After MR scanning, the final Mn concentration of the L-EGCG-Mn buffer solutions (180 samples) was measured via flame atomic absorption spectroscopy (SpectrAA-240FS, Varian, Palo Alto, CA) (wide range [Mn^2+^], 0.02–0.6 mM). Nitric acid was added to decompose the L-EGCG-Mn NPs before detection.

#### Machine and sequences

*In vitro* analysis was performed on a 1.5 T (Aera; Siemens, Healthcare, Erlangen, Germany) MR scanner with a Tx/Rx 15-channel knee coil and a 3 T (Skyra; Siemens Healthcare, Erlangen, Germany) MR system with a Tx/Rx 15-channel knee coil. T_1_ maps were obtained using a series of inversion-recovery sequences with various inversion times (TIs) (Ogg & Kingsley, 2004; Shen et al., 2015). TI = [30, 60, 90, 120, 150, 250, 400, 600, 800, 1200, 1600, 2000, 2400, 2800, and 3200] ms. The repetition time (TR) was equal to 1500 ms + TI. The echo time (TE) was 15 (3 T)/11 (1.5 T) ms. The T_2_ maps were obtained using a protocol involving multi-echo spin-echo sequences (Pintaske et al., 2006; Shen et al., 2019): the TE was between 20 and 600 ms with an interval of 20 ms and the TR was 3000 ms. The following parameters were maintained for all measurements: slice thickness, 5 mm; field-of-view, 80 × 100 mm; matrix, 256 × 256.

#### Calculation of relaxivity

First, the generated DICOM images were analyzed via the ImageJ software package (open source, National Institutes of Health, Bethesda, MD), which was used to place fixed-size circular region-of-interest (ROI) and to automatically calculated mean signal intensities (SIs) within the ROI. ROIs were between 160 and 170 pixels. Second, the relaxivity constants *R*_1_ and *R*_2_ are determined via Equations (1) and (2), respectively, using a developed Data fitting software (Sigma Plot 12.5).

(1) $${SI}_{TI}=A_{1}+{B_{1}\;\text{exp}\;}^{-R1*TI}$$

(2) $${SI}_{TE}=A_{2}\;{\text{exp}}^{-R2*TE}+B_{2}$$

Finally, the *r*_1_ and r_2_ values are obtained using Equation (3) (Fries et al., 2015). Here, *R* (*c*) denotes the relaxivity constant of L-EGCG-Mn at concentration *C* and *R* (0) represents the relaxivity constant of PBS or HSA.

(3) $$r=\left(R_{\left(c\right)}-R_{\left(0\right)}\right)/C$$

### Cellular MR imaging

To evaluate the MR properties of L-EGCG-MN NPs in normoxic and hypoxic cells, CoCl_2_ was used to induce chemical hypoxia (Dubbelboer et al., 2019). Briefly, H22 cells were isolated under sterile conditions and were randomly assigned to either the hypoxia and normoxia groups. Cells were then incubated with medium with or without CoCl_2_ (200 μM) and seeded into six-well plates (1 × 10^7^ cells/well) for 12 h. The cells were then collected and the medium was removed via centrifugation. Thereafter, the cells were washed thrice with PBS to eliminate the residual CoCl_2_. The cells were then resuspended in medium with or without L-EGCG-Mn (1 mM). After being incubated for 4 h, the cells were washed thrice with PBS to eliminate the residual L-EGCG-Mn. Next, agarose gel (1%, 300 μL) was used to resuspend and fix the cells (Zheng et al., 2019). Finally, cellular imaging was carried out using the abovementioned MR system (3 T).

### Animal MRI

H22 tumor-bearing mice were used for animal MRI assessments. Tumor growth occurred in 10 days and the final tumor volume was approximately 100 mm^3^. The tumor volume was measured from vernier caliper and calculated as the length × width (Zhang et al., 2019)×0.5 (Luo et al., 2019). All mice were scanned using a 3.0 T MRI scanner (Skyra; Siemens Healthcare, Erlangen, Germany) with an 8-channel 5-cm Rx custom-design coil. Anesthetized animals were kept warm with a thermostatic electric blanket at 37 °C between imaging sessions. Before imaging, the animals were placed in a prone position. T1 images were acquired pre-injection and at 0.5, 1, 2, and 4 h after the injection of L-EGCG-Mn (6.4 μmol/kg Mn) and Gd-DTPA (6.4 μmol/kg Gd) NPs via the tail vein. The time points were selected based on a previously published study by Mi et al. (2016). The detailed scanning parameters are listed in supplementary Table 1.

Image analysis: signal-to-noise ratio (SNR) and contrast-to-noise ratio (CNR) were measured and calculated by two radiologists based on previous reports (Peng et al., 2018), using Equations (4) and (5), respectively:

(4) $$\text{SNR}=S_{\mathrm{tumor}}/{SD}_{\mathrm{background}}$$

(5) $$\mathrm{CNR}=\left|S_{\mathrm{tumor}\;}{-S}_{\mathrm{tissue}\;}\right|/\sqrt{{{SD}_{\mathrm{tumor}}}^{2}+{{SD}_{\mathrm{tissue}}}^{2}}$$

where *S_tumor_* represents the SI in the ROI placed on a homogeneously enhancing part of the tumor without necrosis and *SD_background_* represents the standard deviation of the background noise. *S_tissue_* represents the SI in the ROI of ipsilateral normal muscle tissue. *SD_tumor_* and *SD_tissue_* represent the standard deviation of the tumor and normal tissue. The ROIs were located in anatomic positions, which were as accurate as possible for the different time points. The above parameters were measured by two experienced radiologists blinded to the CA administered. The average was then obtained for further analysis.

### Fluorescence imaging and *in vivo* distribution of L-EGCG-Mn

DiR (ex = 748 nm, em = 780 nm) can be used to obtain the vivo fluorescence images. Thus, the L-EGCG-Mn NPs were labeled with DiR to investigate their distribution. DiR-labeled L-EGCG-Mn (200 μL, approximately 4 μg DiR per mouse) were injected intravenously. Mice injected with DiR dissolved in PBS were used as a negative control. The mice were euthanized and the heart, liver, spleen, lung, kidneys, tumor, and homolateral inguinal lymph nodes were excised and photographed using a near-infrared fluorescence small animal live imaging system (Pearl Trilogy, LI-COR) at 1, 2, 4, 8, 12, and 24 h post-injection.

### Histological analysis

All mice were euthanized after the last MRI analysis. Afterwards, the specimens, including the major organs (heart, liver, spleen, lung, kidneys), tumor, and homolateral inguinal lymph nodes, were harvested and fixed via immersion in 10% formalin. After paraffin embedding and hematoxylin and eosin staining, the sample sections were evaluated by an experienced histopathologist.

### Statistical methods

All statistical analyses were completed using IBM SPSS 23.0 (Chicago, IL) and GraphPad Prism 7.0 (GraphPad Software, La Jolla, CA). A *p* value<.05 was considered to be statistically significant. The measurement consistency between the two radiologists was tested by calculating the interclass correlation coefficient (ICC). Continuous variables were analyzed using the Kolmogorov–Smirnov test to determine the normality and then, the Student *t*-test (normal distribution) or Mann–Whitney *U*-test (non-normal distribution) was used for comparison.

## Results

### Characterization of L-EGCG-Mn NPs

**Figure 3.**
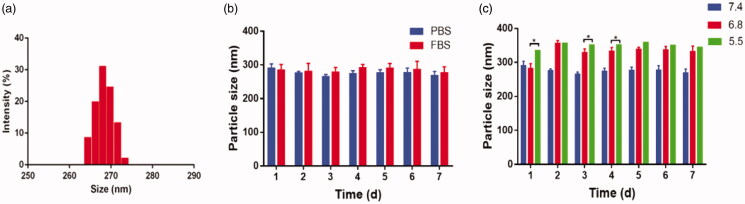
Characterization of L-EGCG-Mn NPs. (a) Particle size of L-EGCG-Mn NPs. (b) The change in particle size of L-EGCG-Mn NPs in phosphate-buffered saline (PBS) and fetal bovine serum (FBS) during a time period of seven days after preparing the solution. (c) The change in particle size of L-EGCG-Mn NPs incubated in buffer solutions with a pH of 7.4, 6.8, or 5.5.

**Figure 4.**
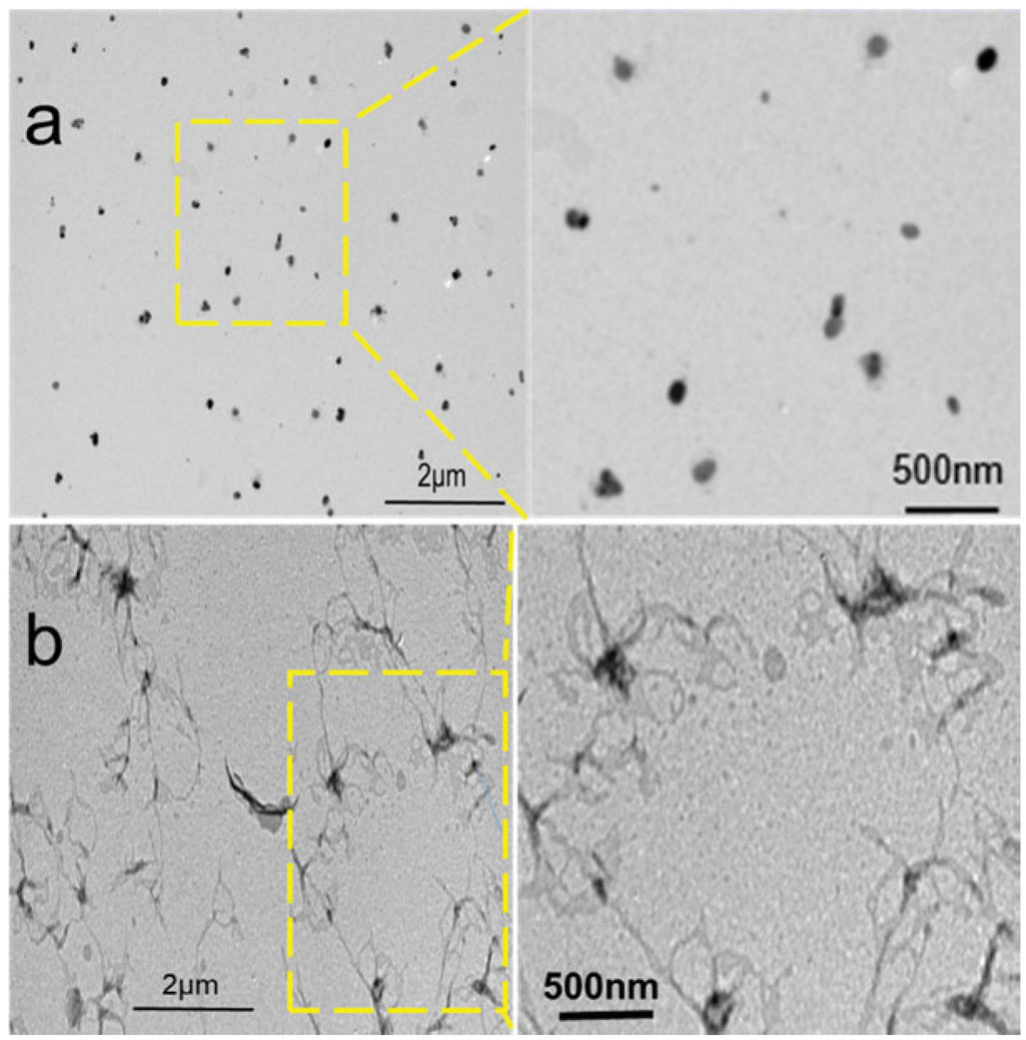
TEM images of L-EGCG-Mn NPs incubated with (a) pH 7.4 buffer solution and (b) pH 5.5 buffer solution.

The particle size of the L-EGCG-Mn NPs was 277.4 ± 5.5 nm (Figure 3(a)) and the zeta potential was −13.56 ± 1.91 mV. Moreover, the particle size change after incubation in both PBS and FBS for 1 week was negligible (Figure 3(b), supplementary figure 1). DLS (Figure 3(c)) showed that the particle size of L-EGCG-Mn NPs incubated with an acidic solution increased over time, with L-EGCG-Mn NPs in a solution with a pH of 5.5 expanding faster than those in a solution with a pH of 6.8. The morphology determined via TEM (Figure 4) revealed that there was a change between L-EGCG-Mn NPs incubated in a solution with a pH of 7.4 or 5.5. In addition, we also observed that the NPs disintegrated in an acidic environment.

### *In vitro* cytotoxicity evaluation

**Figure 5.**
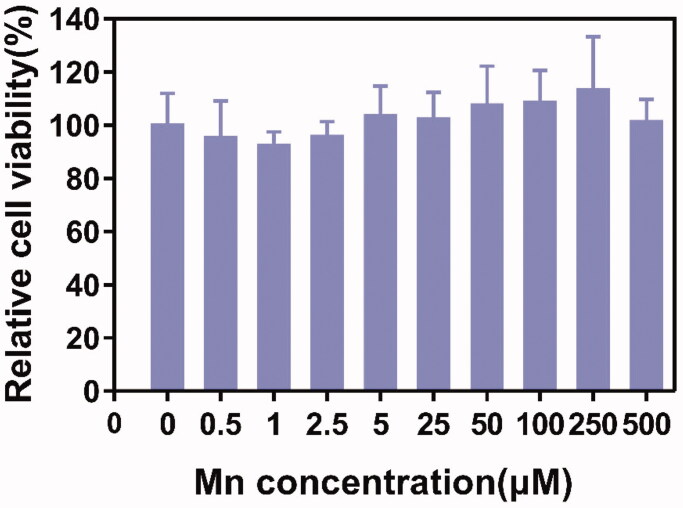
Evaluation of *in vitro* cytotoxicity of L-EGCG-Mn NPs in L929 cells.

L929 cells were used to assess the cytotoxicity of L-EGCG-Mn NPs in normal cells. As shown in Figure 5, cell viability was not markedly reduced when L929 cells were incubated with L-EGCG-Mn NPs for 24 h.

### Relaxivity measurement

**Figure 6.**
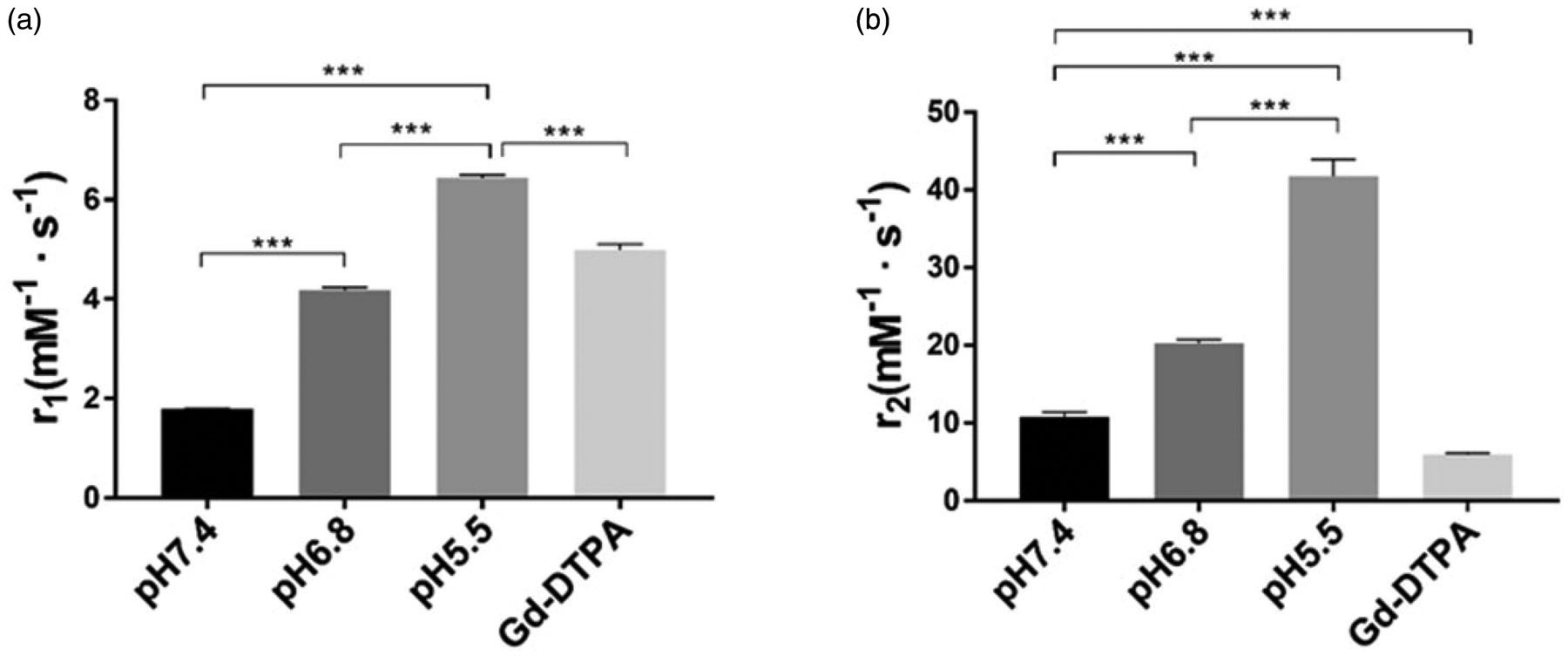
*In vitro* relaxivity. (a, b) The *r*_1_ and *r*_2_ relaxivity of L-EGCG-Mn NPs at different pH values and Gd-DTPA in 3 T MRI. pH 7.4 and 6.8 phosphate buffer solution and pH 5.5 acetate buffer solution were used here. Data are shown as mean ± SD. (*n* = 3), ***p< .001.

The plots of signal vs. TI or TE and of R1 or R2 vs. [Mn] are shown in supplementary figure 2. The relaxivity values of L-EGCG-Mn and Gd-DTPA at 1.5 and 3.0 T are presented in Table 1. For the 3 T MRI, when the pH decreased from 7.4 to 5.5, the *r*_1_ (*r*_2_) value of L-EGCG-Mn NPs increased from 1.79 (10.79) to 6.43 (41.78) mM^−1^·s^−1^ in the buffer solution and from 1.77 (16.1) to 7.23 (42.56) mM^−1^·s^−1^ in HSA. Moreover, there was a significant difference in the relaxivity values between the L-EGCG-Mn NP solutions with different pH (*p*<.001, Figure 6). At pH 5.5, the relaxivity values (*r*_1_ and *r*_2_) of L-EGCG-Mn NPs were found to be higher than that of Gd-DTPA (*p*<.001).

**Table 1.** *In vitro* MRI relaxivities of L-EGCG-Mn and Gd-DTPA.

	1.5 T		3 T	
Contrast agents	*r*_1_ (mM^−1^·s^−1^)	*r*_2_ (mM^−1^·s^−1^)	*r*_1_ (mM^−1^·s^−1^)	*r*_2_ (mM^−1^·s^−1^)
L-EGCG-Mn(+HSA)				
pH 7.4	1.23 ± 0.09 (2.08 ± 0.41)	8.06 ± 0.51 (10.13 ± 1.42)	1.79 ± 0.004 (1.77 ± 0.05)	10.79 ± 0.47 (16.10 ± 1.08)
pH 6.8	2.14 ± 0.37 (2.63 ± 0.71)	13.54 ± 2.09 (14.33 ± 2.48)	4.18 ± 0.04 (5.55 ± 0.06)	20.26 ± 0.38 (26.95 ± 0.37)
pH 5.5	5.93 ± 0.04 (6.45 ± 0.19)	37.59 ± 0.70 (38.09 ± 2.02)	6.43 ± 0.05 (7.23 ± 0.02)	41.78 ± 2.12 (42.56 ± 2.17)
Gd-DTPA	4.38 ± 0.001	4.9 ± 0.03	4.99 ± 0.09	5.99 ± 0.13

MRI: magnetic resonance imaging; Gd: gadolinium; HSA: human serum albumin.

Values are given as mean ± SD in buffered saline (in HSA) at room temperature.

### Cellular MR imaging

**Figure 7.**
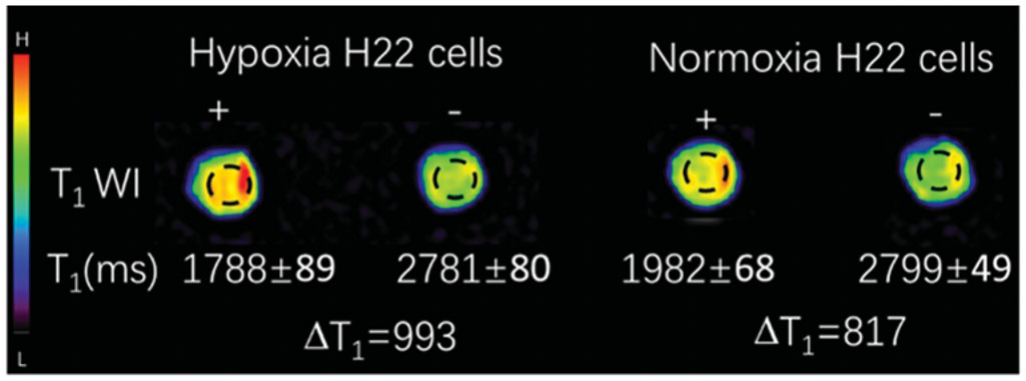
T1-weighted H22 cellular MRI with (+) and without (–) L-EGCG-Mn NPs. The T1 value was obtained from the T1 mapping images. ΔT1 was calculated by subtracting the T1 value for 1 mM Mn from the T1 value for 0 mM Mn.

The shortening of the T1 relaxation time (ΔT1) was calculated by subtracting T1 value for a Mn concentration of 1 mM from the T1 value for 0 mM Mn. After incubation with L-EGCG-Mn NPs for 4 h, the T1 value of hypoxic H22 cells was found to be significantly lower than that of normoxic H22 cells (1788 ± 89 vs. 1982 ± 68 ms, *p*=.041) (Figure 7, supplementary Table 2). Moreover, the ΔT1 of the hypoxia group was shown to be lower than that of the normoxia group (817 vs. 993 ms).

### Animal MRI

**Figure 8.**
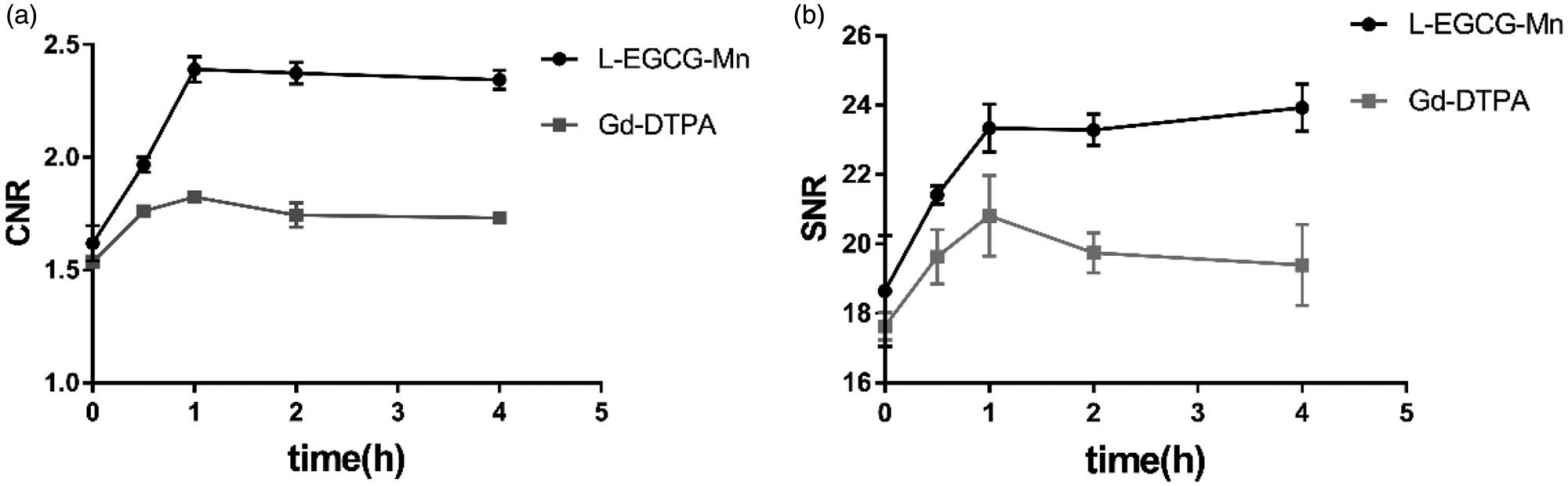
The contrast-to-noise ratio (CNR) and signal-to-noise ratio (SNR) evaluation. (a, b) The trend of CNR and SNR after the administration of L-EGCG-Mn (*n* = 5) and Gd-DTPA (*n* = 3) for T1WI.

**Figure 9.**
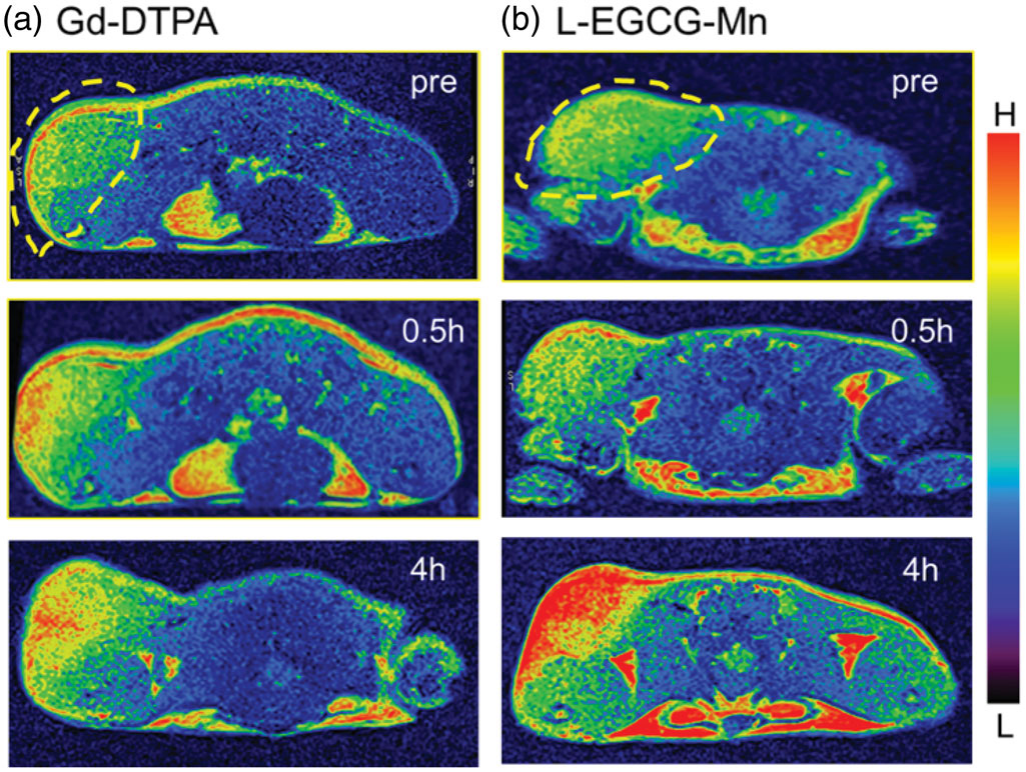
*In vivo* T1WI MR images (3 T) of H22 tumor-bearing mice pre- and post-intravenous injection (i.v.) with Gd-DTPA (a) or L-EGCG-Mn NPs (b). The images are displayed at a window width of 2588 and window level of 1324. L-EGCG-Mn NPs led to a higher enhancement of tumor contrast than Gd-DTPA.

The interobserver agreement for CNR and SNR was excellent (ICC > 0.81, Table 2). For L-EGCG-Mn and Gd-DTPA, the CNR and SNR almost reached their peak at 1 h, followed by a stable high value for the former but a downtrend for the latter (Figure 8). After injection, the average value of CNR and SNR was significantly higher for L-EGCG-Mn NPs than for Gd-DTPA at all acquired timepoints (*p* < .05, supplementary table 3). The classic MRI images of the two mice groups are shown in Figure 9.

**Table 2.** The inter-observer agreement between two radiologists of CNR and SNR.

Parameters	ICC	95% CI
CNR	0.960	0.924–0.979
SNR	0.899	0.806–0.948

ICC: interclass correlation coefficient; CI: confidence interval.

### Fluorescence imaging and *in vivo* distribution of L-EGCG-Mn NPs

**Figure 10.**
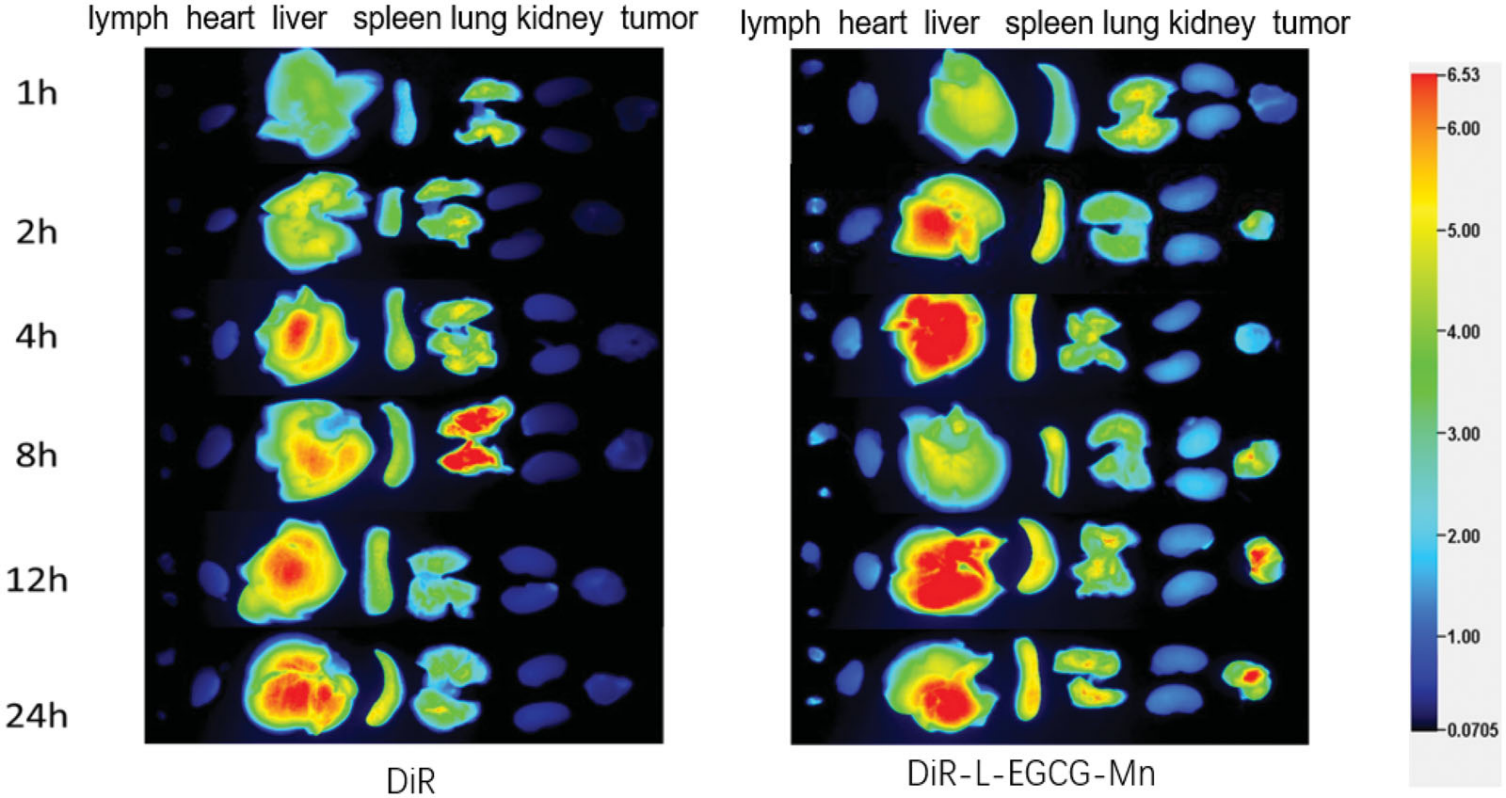
Fluorescence imaging and *ex vivo* distribution of L-EGCG-Mn NPs. *Ex vivo* image of the inguinal lymph nodes, heart, liver, spleen, lungs, kidneys, and tumor of H22 tumor-bearing KM mice injected with DiR and DiR-L-EGCG-Mn NPs for 1, 2, 4, 8, 12, and 24 h.

The DiR-L-EGCG-Mn NPs were found to gradually gather at the tumor site after injection, with the quantity of NPs at the site increasing over time. Moreover, the fluorescence intensity at the tumor site in the DiR-L-EGCG-Mn group was evidently stronger than that in the DiR group (Figure 10). The fluorescence intensity in the inguinal lymph nodes was also stronger in the DiR-L-EGCG-Mn group than that of the DiR group.

### Histological analysis

Histopathological analysis confirmed the presence of hepatoma cells in the tumor samples from all examined animals (supplementary figure 3). After L-EGCG-Mn injection, no appreciable abnormalities were observed in the heart, liver, spleen, lungs, and kidneys. Reactive hyperplasia was observed in the tumor homolateral inguinal lymph node.

## Discussion

Contrast-enhanced MRI fulfills several important medical needs. Therefore, the development and improvement of MRI CAs, especially tumor-targeting agents, is a growing area of study (Adiseshaiah et al., 2013; Gale et al., 2018). The acidic pH environment, mainly caused by lactate, has been extensively proven to be a tumor-specific characteristic (Kanamala et al., 2016). As such, this study aimed to synthesize pH-sensitive NPs to develop a tumor-targeting MRI CA.

We designed a series of experiments to demonstrate the pH-sensitivity of L-EGCG-Mn NPs. First, L-EGCG-Mn NPs were incubated with buffer solutions of a different pH. Analysis of the change in particle size and morphology implied that L-EGCG-Mn NPs were disrupted in low pH environments. Moreover, *in vitro* analysis indicated that the relaxivity of L-EGCG-Mn NPs increased as the pH decreased. The T1 value of L-EGCG-Mn NPs in hypoxic cells was also lower than that found in normoxic cells, which suggests that L-EGCG-Mn NPs are sensitive to pH at the cellular level. These findings supported our hypothesis that a low pH could mediate the disassembly of L-EGCG-Mn NPs and accurately control the release of Mn^2+^ (Li et al., 2010). Thus, we can reasonably assume that L-EGCG-Mn NPs may be applicable for tumor imaging, where the specific acidic environment can serve as relaxation switches activated by pH (Li et al., 2010). Our results also showed that L-EGCG-Mn NPs mainly released Mn^2+^ in the tumor area, which enabled the selective enhancement of tumor tissue and thus increased the contrast between tumor and adjacent normal tissues. In addition, pH-sensitive L-EGCG-Mn NPs may help predict and assess tumor therapeutic outcomes, as an acidic environment is greatly associated with therapeutic response (Swartz et al., 2012; Chang et al., 2015; Pilon-Thomas et al., 2016). However, this hypothesis requires further experimental verification.

Analysis of DiR-L-EGCG-Mn distribution *ex vivo* showed that L-EGCG-Mn NPs possessed excellent tumor-targeting abilities for approximately 24 h post-injection, which could provide a remarkable image-acquisition time window. This is mainly attributable to the enhanced permeability and retention effects (Kim et al., 2018) of NPs. Consequently, we can conclude that high tumor retention and rapid organ clearance makes L-EGCG-Mn NPs an efficient and safe CA.

The *r*_1_ values of L-EGCG-Mn NPs in a buffer solution at 3 T MRI (pH 6.8, 4.18 mM^−1^·s^−1^ and pH 5.5, 6.43 mM^−1^·s^−1^) obtained in this study are comparable to those displayed by HMPB-Mn (Cai et al., 2015) in an aqueous solution at 7 T (pH 5, 7.43 mM^−1^·s^−1^), PEGMnCaP (Mi et al., 2016) in buffer solution at 0.59 T (pH 6.7, 4.27 mM^−1^·s^−1^), and by (UCNP@PFNS/N)@MnCaP (Ji et al., 2019) in an aqueous solution at 7 T (pH 6.8, 4.4 mM^−1^·s^−1^ and pH 5, 6.9 mM^−1^·s^−1^). However, the *r*_1_ value of L-EGCG-Mn NPs in HSA (pH 6.8, 5.55 mM^−1^·s^−1^) is lower than that of PEGMnCaP in HSA (pH 6.7, 15.26 mM^−1^·s^−1^). A possible explanation is that the specific binding between EGCG and HSA limits the binding of Mn^2+^ and HSA (Save & Choudhary, 2018). Interestingly, pH-sensitive CAs, namely L-EGCG-Mn, USMO@MSNs (Wang et al., 2018), and MnO_2_ nanosheets (Chen et al., 2014), are all designed to make the NP core be the most exposed to water molecules in an acidic environment, thereby improving the accessibility of water molecules and Mn^2+^. However, the *r*_1_ value found in this study is higher than that determined for USMO@MSNs (5.61 mM^−1^·s^−1^ in buffer solution, pH 4.5 at 9.4 T MR) and delaminated MnO_2_ nanosheets (4.0 mM^−1^·s^−1^ in buffer solution, pH 4.6 at 3 T MR). This may be due to the fact that the acidic environment can weaken the chelation of EGCG and Mn, thereby substantially accelerating the release of Mn^2+^. Furthermore, the difference in relaxivity may also be associated with the different experimental conditions used, as there are many factors that can influence relaxivity, including solvent type, incubation time, and even temperature (Hao et al., 2012; Goetschi et al., 2014). L-EGCG-Mn NPs also showed pH sensitivity in terms of shortening the T_2_ relaxation time, which is similar to previous studies (Chen et al., 2012).

The high relaxivity of L-EGCG-Mn NPs encouraged us to further explore its MRI performance *in vivo*. We found that the *in vivo* MRI performance of L-EGCG-Mn NPs was comparable to that of the pH-responsive HMPB-Mn (Cai et al., 2015), PEGMnCaP (Mi et al., 2016), and (UCNP@PFNS/N)@MnCaP (Ji et al., 2019). However, the peak SI of HMPB-Mn appeared at approximately 30 min, whereas that of L-EGCG-MN NPs appeared at 1 h. This may be due to the fact that the HMPB-Mn NPs were administrated via intratumor injection. Additionally, L-EGCG-Mn NPs mainly exhibited a homogeneous enhancement, which is different from the selective high strengthening of PEGMnCaP and (UCNP@PFNS/N)@MnCaP. The significantly lower concentration of Mn used in this study (6.4 μmol/kg) may be a potential explanation, as well as the use of a different tumor type (H22 tumors) when compared to existing studies (PEGMnCaP: 225 μmol/kg based on Mn for C26 tumors; UCNP@PFNS/N @MnCaP: 750 μmol/kg based on Mn for HepG2 tumors).

The above-mentioned Mn-based CAs are still in the basic research stage. Therefore, although they are useful as a reference for comparison against L-EGCG-Mn NPs, it is also important to compare L-EGCG-Mn NPs with clinically used CAs. Thus, the first approved extracellular Gd chelate, namely Gd-DTPA, was used to compare the properties of L-EGCG-Mn NPs. *In vitro* experiments showed that the T_1_ and T_2_ relaxivity of L-EGCG-Mn NPs were significantly higher than those of Gd-DTPA. Moreover, in the H22 tumor-bearing mice model, L-EGCG-Mn NPs led to improved SNR and CNR when compared to Gd-DTPA in T1WI. In addition to the pH sensitivity of L-EGCG-Mn NPs, it is also possible that this may be due to the fact that Gd-DTPA has only one water molecule coordination site due to the DTPA ligand forming a stable structure around Gd^3+^ (Cai et al., 2015).

Additionally, histochemical analysis showed that the inguinal lymph nodes exhibited inflammatory hyperplasia, possibly as an effect of the neoplasm. Interestingly, analysis of *in vivo* distribution showed that the aggregation of L-EGCG-Mn NPs in the lymph nodes increased with time. This phenomenon may be explained by the acidic microenvironment created due to inflammatory hyperplasia (Gallagher et al., 2008). Thus, the relationship between inflammatory tissues and Mn NPs needs further study.

## Conclusions

This study developed EGCG-Mn NPs enveloped with phospholipids and thus obtained NPs with a good safety profile and high pH sensitivity that may be used as MRI CAs. L-EGCG-Mn NPs could respond to tumor-related pH changes and, therefore, may serve as a potential tumor-targeting CA due to its good MRI properties in both a hypoxic cell model and H22 tumor-bearing mice model.

## Supplementary Material

Supplemental Material
