# Supplementary material for: L-EGCG-Mn nanoparticles as a pH-sensitive MRI contrast agent
Source: Drug Deliv. 2020 Dec 26;28(1):126–35. doi: 10.1080/10717544.2020.1862363 (PMC7782420; doi:10.1080/10717544.2020.1862363)
Supplement: Supplemental Material [file IDRD_A_1862363_SM1411.docx]

Supplementary table 1. Detailed parameters of the acquired sequences

|  | TR, ms | TE, ms | Flip Angle, Degrees | No. Averages | FOV, mm^2^ | Matrix | Pixel Size mm^2^ | Slice Thickness mm | |
| --- | --- | --- | --- | --- | --- | --- | --- | --- | --- |
| T1WI | 500 | 17 | 160 | 6 | 40*40 | 256*205 | 0.156*0.156 | 1 |  |
| T2WI | 3990 | 87 | 160 | 6 | 40*40 | 192*173 | 0.208*0.208 | 1 |  |


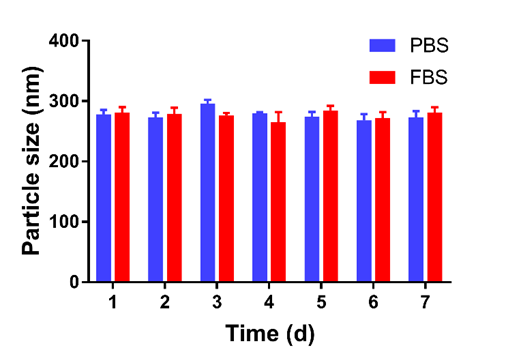


Supplementary figure 1. The change of particle size of DiR-labeled L-EGCG-Mn in phosphate-buffered saline (PBS) and fetal bovine serum (FBS) during seven days after it was prepared.


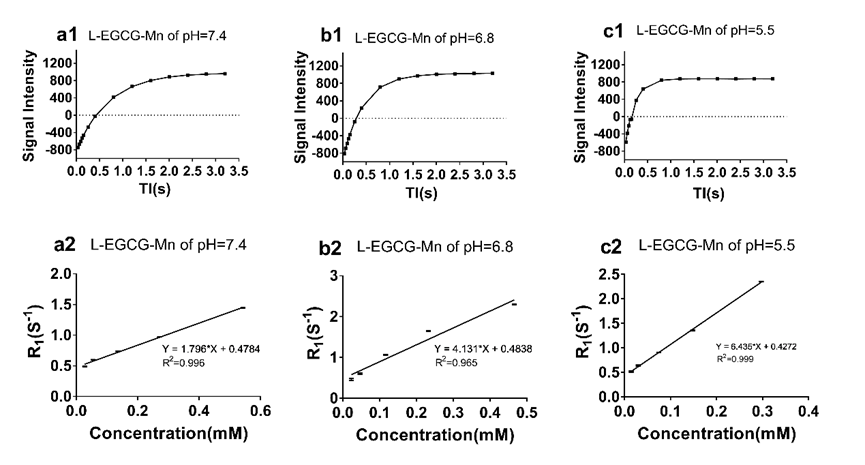


Supplementary figure 2A: Plots of Signal vs TI and plots of R_1_ vs [Mn] obtained at 3T MR scanner. (a1) and (a2) were in pH= 7.4 PBS, (a1) was obtained at a Mn concentration of 0.543 mM. (b1) and (b2) were in pH= 6.8 PBS，(b1) was obtained at a Mn concentration of 0.466 mM. (c1) and (c2) were in pH= 5.5 PBS, (c1) was obtained at a Mn concentration of 0.297 mM.


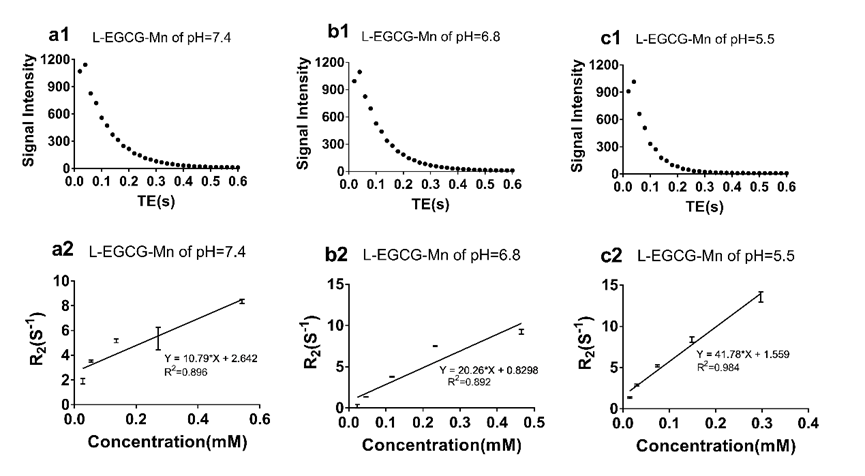


Supplementary figure 2B: Plots of Signal vs TE and plots of R_2_ vs [Mn] were obtained at 3T MR scanner. (a1) and (a2) were in pH= 7.4 PBS, (a1) was obtained at a Mn concentration of 0.543 mM. (b1) and (b2) were in pH= 6.8 PBS, (b1) was obtained at a Mn concentration of 0.466 mM. (c1) and (c2) were in pH= 5.5 PBS, (c1) was obtained at a Mn concentration of 0.297 mM.

Supplementary table 2: T1-weighted H22 cellular MR imaging with (+) and without (-) L-EGCG-Mn.

|  | T1 value of (-)L-EGCG-Mn | | | T1 value of (+)L-EGCG-Mn | |
| --- | --- | --- | --- | --- | --- |
|  | Normoxia cells | | Hypoxia cells | Normoxia cells | Hypoxia cells |
| Sample1 | 2640.61 | 2750.27 | | 1904.39 | 1840.26 |
| Sample2 | 2763.95 | 2724.79 | | 2034.17 | 1685.48 |
| Sample3 | 2834.46 | 2838.49 | | 2007.62 | 1839.24 |
| mean±SD | 2799±49 | 2781±80 | | 1982±68 | 1788±89 |

T1 value was obtained from T1mapping images.

Supplementary table 3. CNR and SNR comparison of the different CAs for all examination time points

|  |  |  |  | | T1WI |  |  |
| --- | --- | --- | --- | --- | --- | --- | --- |
|  |  | Pre | 0.5h | | 1h | 2h | 4h |
| CNR | L-EGCG-Mn | 1.62(0.07) | 1.97(0.03) | | 2.39(0.06) | 2.37(0.05) | 2.34(0.04) |
|  | Gd-DTPA | 1.54(0.03) | | 1.76(0.01) | 1.82(0.02) | 1.74(0.05) | 1.73(0.02 ) |
|  | *p* | 0.108 | **＜0.001** | | **＜0.001** | **0.036^a^** | **0.036^a^** |
| SNR | L-EGCG-Mn | 21.87(3.33) | 21.41(0.26) | | 23.34(0.69) | 23.29(0.45) | 23.92(0.68) |
|  | Gd-DTPA | 20.81(1.15) | 19.63(0.78) | | 20.81(1.15) | 19.74(0.57) | 19.39(1.17) |
|  | *p* | 0.623 | **0.007** | | **0.015** | **0.036^a^** | **0.036^a^** |

Values are given as mean (SD); ^a^Mann–Whitney U-test;

CNR indicates contrast-to-noise ratio; SNR, signal-to-noise ratio; Gd, gadolinium; CAs, contrast agents.


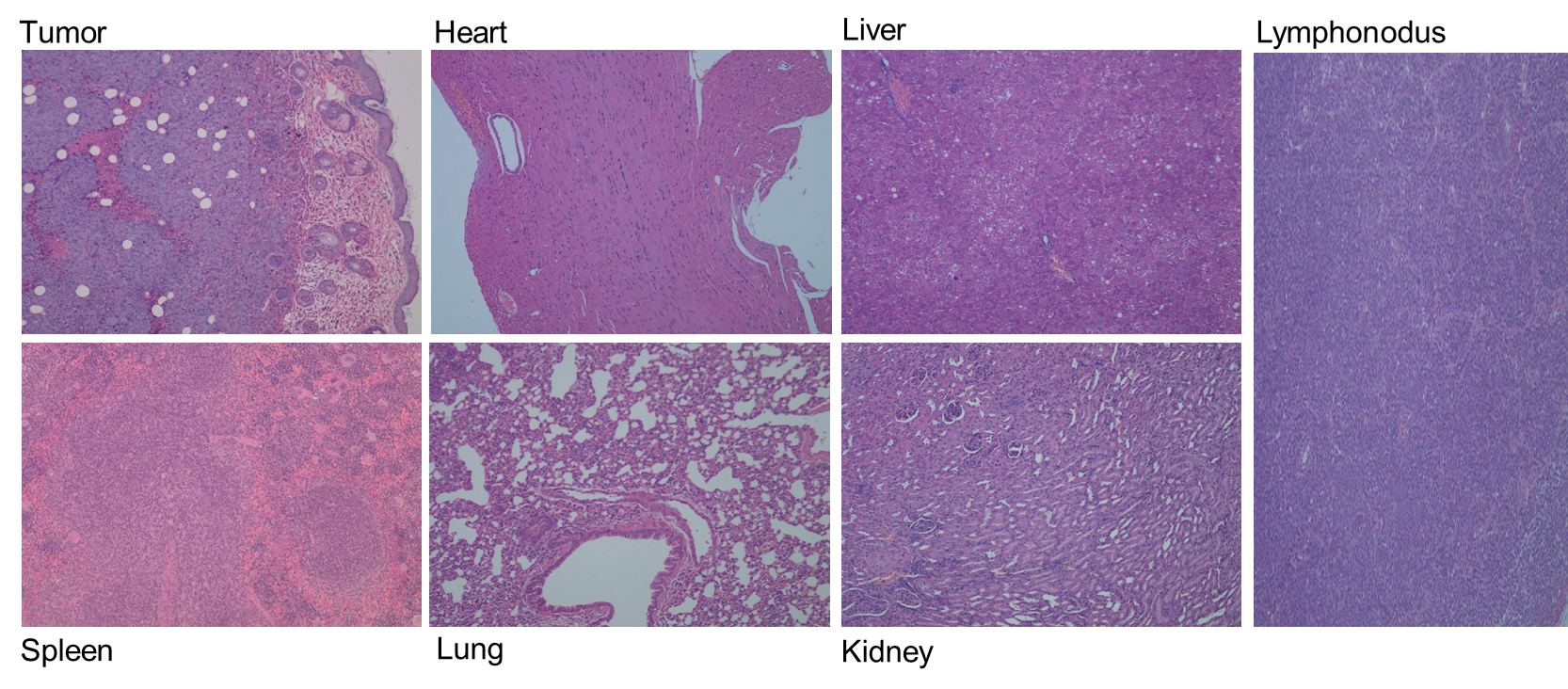


Supplementary figure 3. Mice pathological images of tumor, main organs and lymphonodus, after 4 h of L-EGCG-Mn injection (*100).
